# Paratyphoid a, with Relapse Showing Eberth Bacillus

**Published:** 1917-08

**Authors:** 


**Paratyphoid A, With Relapse Showing Eberth Bacillus.** P. Gerard and Fenestre, Le Prog. Med., Feb. 24, report a case, the diagnosis being determined in the attempt to explain certain contradictory serum reactions. They give the general warning that bacteriologic identification of the similar fevers due to typhoid and the two paratyphoid bacilli may be fallacious on account of one bacillus assuming the ascendency in cultures.

